# Detection of neuroendocrine tumours in the small intestines using contrast-enhanced multiphase Ga-68 DOTATOC PET/CT: the potential role of arterial hyperperfusion

**DOI:** 10.2478/raon-2014-0012

**Published:** 2014-04-25

**Authors:** Nils F. Schreiter, Martin Maurer, Ulrich-Frank Pape, Bernd Hamm, Winfried Brenner, Vera Froeling

**Affiliations:** 1 Department of Nuclear Medicine, Charité, Berlin, Germany; 2 Department of Radiology, Charité, Berlin, Germany; 3 Department of Gastroenterology, Charité, Berlin, Germany

**Keywords:** Ga-68 DOTATOC PET/CT, small intestine, neuroendocrine tumors, arterial phase CT

## Abstract

**Background:**

Interpretation of small intestinal neuroendocrine tumours (NETs) by Ga-68 DOTATOC PET/CT can be difficult. The potential benefit of arterial hyperperfusion for the detection of NETs was evaluated.

**Methods:**

Between 2006 and 2009, 320 consecutive Ga-68 DOTATOC PET/CT examinations, performed for NETs, revealed 40 lesions suggesting intestinal NETs in 25 patients. Two groups of lesions were distinguished: epigastric lesions evaluable in the arterial and venous CT scan (Group 1) and hypogastrial lesions evaluable in the venous CT scan only (Group 2). Lesions were jointly rated by two radiologists and a nuclear medicine physician. Maximum standard uptake values (SUVmax) of lesions and background were assessed. The reference standard was histology (available for 28 lesions) or follow-up (for a mean of 22.9 months).

**Results:**

PET detected all suspicious lesions but was false positive in 3 lesions. In Group 1 the arterial scan performed significantly better than the venous scan (p = 0.008). Diagnostic performance was better in Group 1 than in Group 2 (p < 0.001). SUVmax of true positive lesions were significantly higher than background SUVmax (p < 0.001) and SUVmax of false positive lesions (p = 0.005).

**Conclusions:**

The arterial phase of multiphase Ga-68 DOTATOC PET/CT might improve the localization of intestinal NETs and, thereby, improve the overall diagnostic accuracy of this modality in the assessment of intestinal NETs by adding information about lesion perfusion not available when only venous CT is performed.

## Introduction

Neuroendocrine tumours (NETs) are a heterogeneous group of neoplasms of neuroendocrine origin.^1^ The annual incidence is low at 1-2/100 000 people.^2^ Two thirds of all NETs are found in the gastrointestinal tract including the pancreas and the hepatobiliary system (very rare).^3^ An increasing incidence of these tumours has been detected over the last 20 years, which is partially attributable to advances in diagnostic modalities.^4^,^5^ A promising approach for diagnosis and therapy is somatostatin receptor targeting.

Ga-68-DOTA(0)-Phe(1)-Tyr(3)-octreotide (Ga-68 DOTATOC) is a somatostatin analogue with affinity for somatostatin receptor 2 (SSTR-2) and has a higher sensitivity and specificity than the current gold standard, single photon emission computed tomography (SPECT) with the somatostatin analogue In-111 diethylene triamine pentaacetic acid octreotide (In-111 DTPA octreotide).^6^ Ga-68 DOTATOC positron emission tomography (PET) has been found to detect significantly more lesions than In-111 DTPA octreotide SPECT.^7^ A major advantage of Ga-68 DOTATOC PET/CT is that it combines somatostatin receptor imaging with a full contrast-enhanced multiphase computed tomography (CT) scan. While somatostatin receptor imaging has high specificity, the CT scan provides good image resolution and enables a dynamic evaluation following contrast medium administration, which improves the detection of small NETs.^8^,^9^ A drawback of CT is that it relies on lesion size and enhancement characteristics for lesion characterization, which has low specificity.^10^

Ruf *et al.* have shown that multiphase Ga-68 DOTATOC PET/CT has a significant impact on the patient’s management with PET and CT providing complementary information.^11^ Other authors also report an impact on the patient’s management.^12^,^13^ In the study by Ruf *et al*., CT was significantly superior to PET in detecting the small number of 17 intestinal NETs, which the authors attributed to the difficulty in differentiating between physiologic intestinal accumulation of Ga-68 DOTATOC and abnormal accumulation of this tracer in intestinal NETs.^11^ For liver metastases of NETs and for NET primaries in the pancreas the value of multiphase CT has already been described.^14^–^20^ As far as we know the value of arterial hyperperfusion of NETs in the small intestines and how it can be exploited in diagnostic imaging has never been investigated before.

The aim of the present study is to investigate whether contrast-enhanced multiphase PET/CT in general, and the arterial phase in particular, have an added benefit for the detection of NETs in the small intestines.

## Patients and methods

We retrospectively analyzed the records of 320 Ga-68 DOTATOC PET/CT examinations performed at our department from 2006 to 2009 for the diagnostic evaluation of patients with NETs. An experienced specialist in nuclear medicine identified 25 patients (12 males, 13 females; age range: 35–79 years; mean: 56.5 years, median: 56 years) with 40 findings suggesting primary NETs in the small intestine. There were 16 patients with cancer of unknown primary (CUP) in whom the examination was performed to search for the primary tumour and 9 patients who underwent PET/CT for staging. Only one examination per patient was included in this study.

Ga-68 DOTATOC was prepared by our radio-chemist as described by Zhernosekov *et al*.^21^ The PET scans were acquired 45 min to 60 min after injection of approximately 100–120 MBq of Ga-68 DOTATOC.

The examinations were performed on a 16-row PET/CT system (Biograph 16; Siemens AG, Erlangen, Germany). In 22 patients, CT was performed using a triple-phase protocol with CareDose4D (230 mAs eff., 120 kV) and 70–100 ml of IV contrast medium (Ultravist 370; Bayer Schering Pharma, Berlin, Germany). The delay was 24 s for the arterial bolus and 45 s for the portal-venous phase, both obtained with bolus tracking. During each phase, an upper abdominal scan with a slice thickness of 16 × 0.75 mm was acquired. For the venous phase, the delay was 70 s and 16 × 1.25 mm slice thickness was acquired. In 3 examinations, CT was performed as a low-dose CT without contrast medium (40 mAs eff/120 kV).

The PET scans were acquired over 5–6 bed positions of 3 minutes each, covering the area from the base of the skull to the upper thigh. PET images derived from a 168 × 168 matrix acquisition were iteratively reconstructed with scatter correction using the ordered subset expectation maximization technique (5 iterations, 8 subsets). Attenuation correction was based on an attenuation map generated from the whole-body venous-phase CT scan or the low-dose CT scan. No radiopaque oral contrast medium was given as it may degrade PET images.^22^

Two experienced radiologists and one nuclear medicine physician first interpreted PET and CT alone and then simultaneously. Lesions were classified into three categories: suspicious, nonsuspicious, and suspicious in conjunction with PET (hyperperfusion). The 3 PET/CT examinations without contrast medium administration were excluded from this analysis. The remaining lesions were assigned to one of two groups: lesions evaluable in the venous and arterial CT scan (Group 1) and lesions evaluable in the venous CT scan only (Group 2).

Maximum standard uptake values (SUVmax) were calculated at a Leonardo workstation (Siemens AG, Erlangen, Germany). A region of interest (ROI) was drawn around the suspicious lesion to assess its SUVmax. An approximate average background SUVmax was calculated as the mean of 5 ROIs placed in bowel segments not suspicious for NET.

The histopathologic diagnosis (available for 28 lesions) or the results of another diagnostic modality such as endoscopy and/or follow-up imaging (for 12 lesions) for a mean of 22.9 months (median 14.5; range: 6–52 months) were used as the standard of reference.

### Statistical analysis

Data were collected using Excel (Microsoft®, Windows®XP). The statistical analysis was performed with PASW 18 (IBM, USA). The Wilcoxon rank-sum test was used to assess the level of significance for the differences between lesion SUVmax and background SUVmax.

A p-value < 0.05 was considered significant. Differences between SUVmax and the SUVmax lesion-to-background ratio of true positive and false positive lesions were analysed using the Mann-Whitney U-test. A receiver operating characteristic (ROC) analysis of SUVmax and SUVmax lesionto-background ratio was performed. The difference between no lesion correlate (nonsuspicious) and a lesion correlate (suspicious + suspicious in conjunction with PET) in Group 1 was assessed by the McNemar test, and the differences between the overall diagnostic performance in Group 1 and Group 2 by the Fisher`s exact test.

The institutional ethics review board approved this retrospective study.

## Results

**FIGURE 1.**
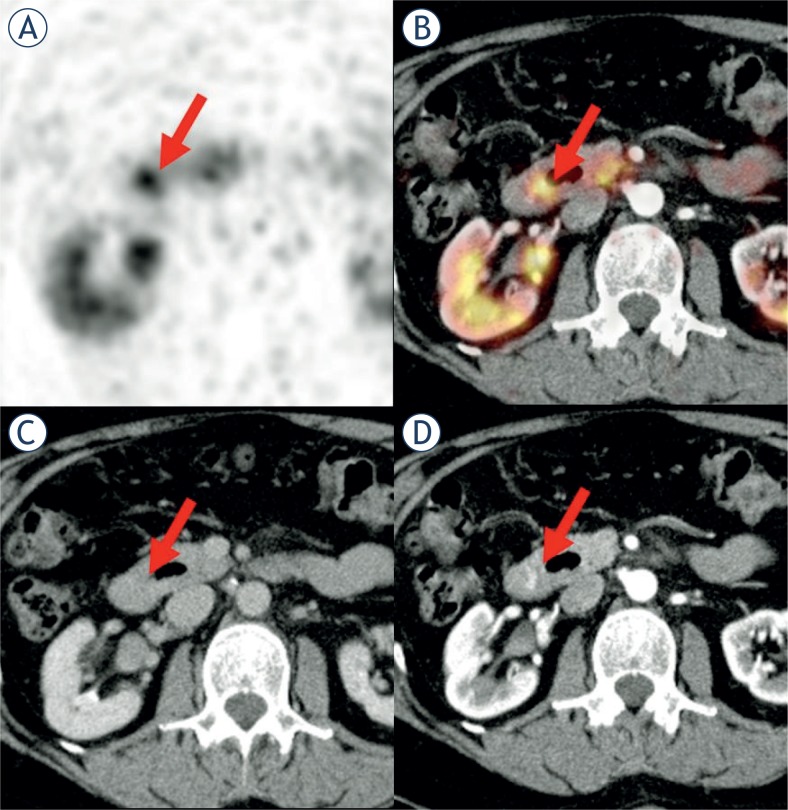
Suspicious Ga-68-DOTATOC PET lesion (#2) **(A)** located in the duodenum **(B)** without a clear correlate in the venous-phase CT **(C)**, but arterial hyperperfusion **(D)**.

**FIGURE 2.**
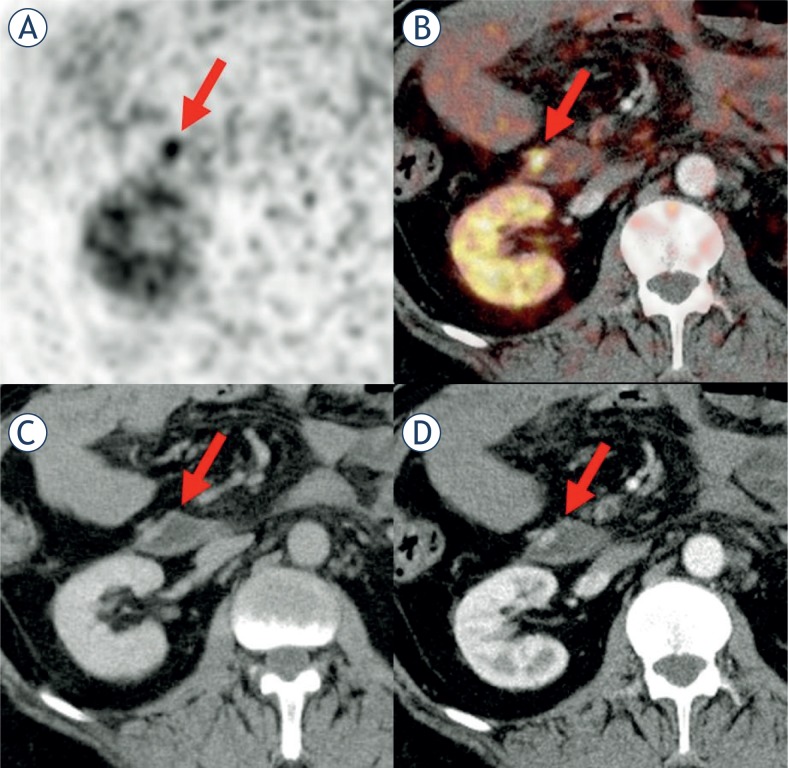
Another suspicious Ga-68-DOTATOC PET lesion (#11) **(A)** located in the duodenum **(B)** without a clear correlate in the venous-phase CT **(C)**, but arterial hyperperfusion **(D)**.

**FIGURE 3.**
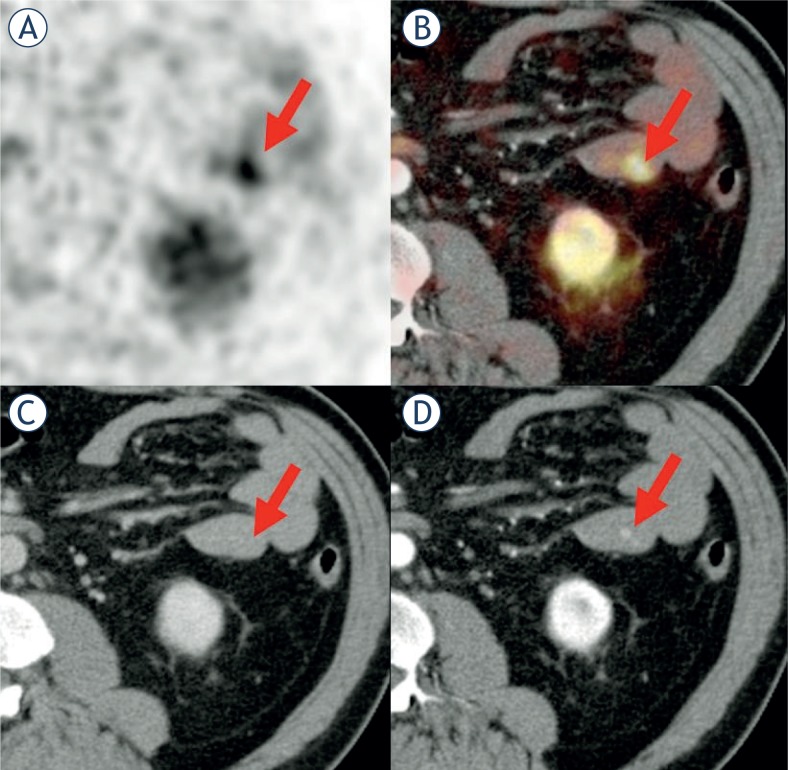
Patient with biopsy-proven NET liver metastases. The Ga-68-DOTATOC focus (#7a) **(A)** in the jejunum **(B)** has no correlation in the venous-phase CT **(C)**, while a lesion is clearly detectable in the arterial-phase CT **(D)**.

**FIGURE 4.**
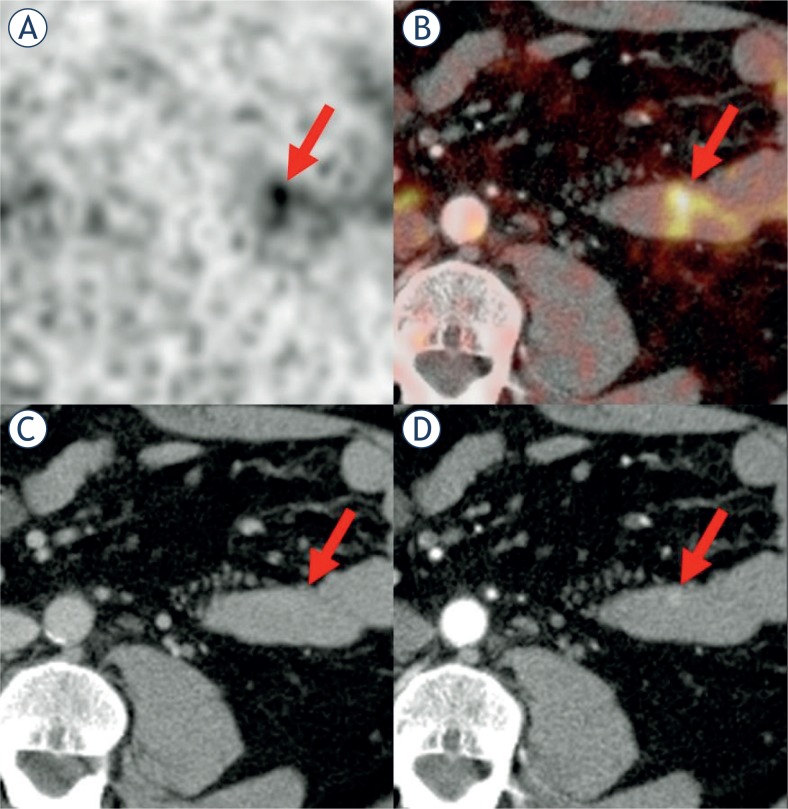
The same patient as in Figure 3 had another Ga-68-DOTATOC-positive focus (#7b) **(A)** more distal in the jejunum without a correlate in the venous-phase CT **(C)** but matching a lesion visible in the arterial-phase CT **(D)**. This lesion was not detected during surgery; however, it was definitely confirmed by histopathology.

Seven of 25 patients with suspected NETs of the small intestines had multifocal lesions, resulting in a total of 40 suspected intestinal NETs. An overview of all lesions is presented in Table 1. Three lesions (in 2 patients) of the 40 suspicious lesions were subsequently classified as false-positive based on the reference standard. There were no CT abnormalities in either the arterial or venous phase in these cases. In Group 1, 14 (in 13 patients) of the PET-positive lesions could be evaluated on both arterial and venous CT scans (Figures 1–4). The results are summarized in Table 2. The arterial CT scans detected 3 lesions (21.4%) when interpreted alone and 8 lesions (57.1%) in conjunction with PET, while 3 lesions (21.4%) could not be detected at all. The venous CT scan detected only 3 lesions (21.4%), while 11 lesions (78.6%) were rated as non-suspicious. The venous CT in conjunction with PET did not offer any new information about lesions. In conjunction with PET the arterial CT scan was significantly superior to the venous CT scan (p-value = 0.008). In Group 2, 17 of the PET-positive lesions (in 7 patients) could only be evaluated on venous-phase CT scans: only 2 (11.8%) of the 17 lesions were suspicious at venous-phase CT, the remaining lesions appeared normal (n = 15, 88.2%). The patient group with evaluable lesions in the arterial and venous CT scan (Group 1) was significantly superior to the group with evaluable lesions in the venous CT only (Group 2) regarding the sensitivity (p-value > 0.001) for NET lesions. Only 6 PET-positive lesions (in 3 patients) could be judged in the low-dose CT scan none of them had a correlate in the low-dose scan.

**FIGURE 5.**
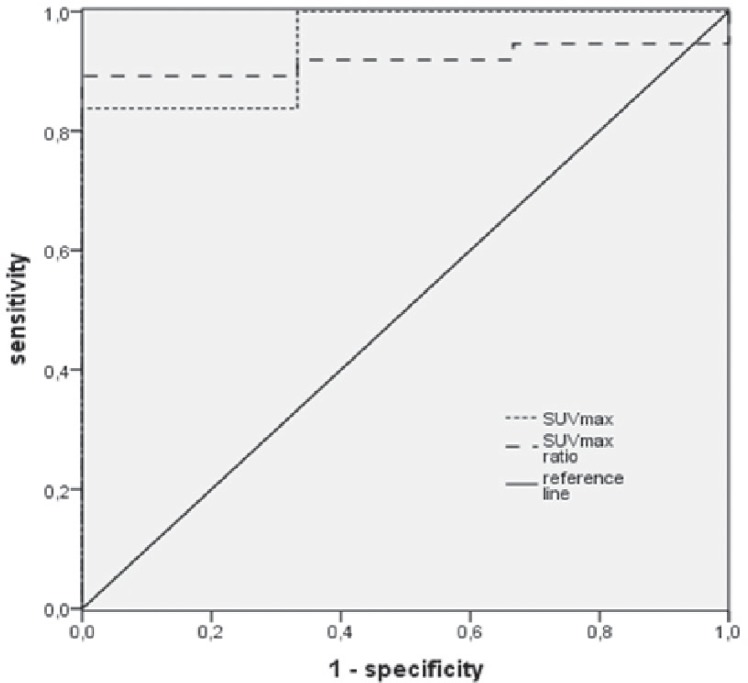
ROC analysis of lesion SUVmax and lesion-to-background SUVmax ratios.

The mean SUVmax of true positive lesions (n = 37) was 18.48 (median: 14.9; range: 5.5–64.6). The mean SUVmax lesion-to-background ratio for 23 patients was 3.32 (median: 3.2; range: 1.7–5.1). The false positive lesions (n = 3) had SUVmax of 7.5, 3.6, and 4.5. The lesion SUVmax and background SUVmax were significantly different (p-value > 0.001). The SUVmax and the lesion-to-background SUVmax ratio lesion/background of true positive lesions and false positive lesions were also significantly different (p-value = 0.005 and 0.011). In ROC analysis the area under the curve (AUC) of lesion SUVmax was 0.946 and the AUC of lesion-to-background SUVmax was 0.919 (Figure 5).

## Discussion

Our results suggest that many primary NETs in the small intestines display not only an increased expression of somatostatin receptors, which was shown to be very effective for the diagnosis of intestinal NETs, but also frequently arterial hyperperfusion. The arterial phase and not the venous phase appears to be beneficial in detecting NETs of the small intestines using multiphase Ga-68 DOTATOC PET/CT.

Arterial hyperperfusion has been reported to characterize both metastasis from NETs and primary NETs.^18^–^20^ In our patient population, only a few NETs were identified in venous-phase CT; this applies to both hypogastric NETs that could be evaluated in the venous phase only and epigastric NETs that could be evaluated in arterial and venous phases. While venous-phase CT mainly relies on lesion size, the arterial phase can add information on perfusion. A study by Versari *et al*. investigated the detection of duodenopancreatic NETs using endoscopic ultrasound (EUS), Ga-68 DOTATOC PET, and CT. They report a comparable accuracy for each of these modalities alone, concluding that their combination may allow an optimal preoperative diagnosis.^23^ While the 23 NETs in 19 patients investigated by Versari *et al*. also included pancreatic lesions, we only investigated lesions in the small intestines. Moreover, Versari *et al*. did not analyze arterial and venous CT scans separately. In a study evaluating the role of Ga-68 DOTATOC with a triple-phase CT protocol Ruf *et al*. detected gastrointestinal lesions with PET only.^24^ Regarding gastrointestinal lesions a drawback of this study is that only 2 NET lesions were analyzed. Our finding that PET appears to be more appropriate than CT for the detection of intestinal NETs is in disagreement with another study of Ruf *et al*. which investigated the role of Ga-68 DOTATOC PET/CT for the therapy management.^11^ In most of our cases, arterial hyperperfusion can be seen in conjunction with PET reading only, because the lesions are very small and the enhanced area is hard to differentiate from the inhomogeneous appearance of bowel loops. However, PET requires CT for correct localization and characterization of intestinal lesions.

Reliable characterization of NETs of the small intestines is difficult on the basis of abnormal Ga-68 DOTATOC PET findings alone.^11^ This situation is mainly attributable to physiologic Ga-68 DOTATOC accumulation in the intestine and the fact that the tracer typically shows an inhomogeneous distribution. For these reasons, findings in organs with physiologic tracer accumulation should be interpreted with caution.^8^ Our results suggest that the SUVmax can help to decide whether a lesion is malignant or benign. Nevertheless, we think that caution is in order in suggesting a threshold. Our statistical analysis relies on only 3 false positive lesions and the background SUVmax is only an approximation averaged over 5 ROIs. SUVmax in normal intestinal tissue may be much higher than an averaged background SUVmax. Another reason for using background SUVmax with caution is that the AUC of lesion SUVmax is slightly higher than the AUC of the lesion-to-background SUVmax ratio. However, very high SUVmax are strong clues for NETs.

Our patient population is biased towards small lesions. Larger NETs of the small intestines are easier to detect and have typically been identified by other diagnostic tests such as endoscopy or CT before PET/CT is performed. In contrast, most of the patients we investigated here had CUP, which means that primary intestinal tumours are very small and have not been detected by other diagnostic modalities before. Detection of a Ga-68 DOTATOC positive lesion is of course easier when the target-to-background ratio is high as opposed to a low ratio as is typical in a small lesion against a heterogeneous background.

Mainly in case of inhomogeneous tracer distribution contrast-enhanced multiphase CT can help to overcome the limitations of the diagnostic performance of Ga-68 DOTATOC PET. The combination of hyperperfusion and increased somatostatin receptor expression increases the detection of NETs of the intestine and should improve diagnostic confidence. A reader who notices a Ga-68 DOTATOC focus that he or she cannot classify with confidence can additionally look at the arterial phase CT images. Conversely, a hyperperfused lesion at CT can be verified by checking the corresponding PET scan. PET/CT, therefore, should increase both accuracy and diagnostic confidence.

Normal intestinal motility may make it difficult to match a nuclide focus with the correct intestinal loop on CT. Here, arterial hyperperfusion may also be helpful and improve localization with endoscopy in case the imaging result is clear and no further testing is required for surgical resection. Studies have reported promising results for somatostatin PET/CT.^7^,^11^,^13^,^25^ An optimized PET/CT protocol including an intestinal scan during arterial enhancement might improve the detection of primary NETs in this location even further. The protocol for detecting intestinal NETs may be further improved by a negative oral contrast agent such as water and butylscopolamine administration for reducing intestinal motility.

## Conclusions

Our results indicate that a considerable number of intestinal NETs may demonstrate arterial hyperperfusion. A Ga-68 DOTATOC PET/CT protocol for the evaluation of intestinal NETs should include an arterial phase CT scan of the bowel loops, especially when it is used to search for primary tumours in patients with CUP. Together with foci of high SUVmax, visual interpretation of arterial hyperperfusion is a strong clue for NET lesions in the small intestines and can be helpful for image interpretation and lesion localization.

## Figures and Tables

**TABLE 1.** Overview of all suspicious lesions of the small intestine (n = 40/25 patients)

**n=40**	**Number of evaluable lesions**	**TP**	**TN**	**FP**	**FN**
PET	40	37	0	3	0
CT, multiphase	16	See Table 2	2	0	See Table 2
CT, ven. phase only	18	2	1	0	15
CT low dose	6	0	0	0	6

FN = false negative; FP = false positive; TN = true negative; TP = true positive

**TABLE 2.** Comparison of the performances of PET and arterial- and venous-phase CT

**N = 14**	**#1**	**#2**	**#3**	**#4**	**#5**	**#6**	**#7a**	**#7b**	**#8**	**#9**	**#10**	**#11**	**#12**	**#13**
PET	+	+	+	+	+	+	+	+	+	+	+	+	+	+
CT, art. phase	-	c	c	c	c	+	c	c	+	-	c	c	+	-
CT, ven. phase	-	-	-	-	-	+	-	-	+	-	-	-	+	-
SUVmax	23.4	43.6	5.9	43.7	31.5	6.2	9.0	5.5	64.6	9.5	10.3	11.3	58.0	14.1
Localisation	ile	duo	ile	duo	duo	duo	jej	jej	duo	duo	jej	duo	ile	duo
Proceeding after PET/CT	OP	OP	FU 36m	FU 37m	OP	OP	OP	OP	OP	OP	FU 14m	OP	OP	FU 52m

+ = suspicious lesion; - = nonsuspicious lesion; c = suspicious CT lesion in combination with PET (hyperperfusion) for true positive NET lesions (n = 14/13 patients) according to the reference standard; duo = duodenum; FU = follow-up; ile = ileum; jej = jejunum; m = months; OP = operation

## References

[1] Kocha W, Maroun J, Kennecke H, Law C, Metrakos P, Ouellet JF (2010). Consensus recommendations for the diagnosis and management of well-differentiated gastroenterohepatic neuroendocrine tumours: a revised statement from a Canadian National Expert Group. Curr Oncol.

[2] Modlin IM, Lye KD, Kidd M (2003). A 5-decade analysis of 13,715 carcinoid tumors. Cancer.

[3] Modlin IM, Latich I, Zikusoka M, Kidd M, Eick G, Chan AK (2006). Gastrointestinal carcinoids: the evolution of diagnostic strategies. J Clin Gastroenterol.

[4] Bornschein J, Kidd M, Malfertheiner P, Modlin IM (2008). Gastrointestinal neuroendocrine tumors. Dtsch Med Wochenschr.

[5] Slavec ZZ, Gaberscek S, Slavec K (2012). The development of nuclear medicine in Slovenia and Ljubljana: half a century of nuclear medicine in Slovenia. Radiol Oncol.

[6] Kowalski J, Henze M, Schuhmacher J, Macke HR, Hofmann M, Haberkorn U (2003). Evaluation of positron emission tomography imaging using [68Ga]-DOTA-D Phe(1)-Tyr(3)-Octreotide in comparison to [111In]-DTPAOC SPECT. First results in patients with neuroendocrine tumors. Mol Imaging Biol.

[7] Buchmann I, Henze M, Engelbrecht S, Eisenhut M, Runz A, Schafer M (2007). Comparison of 68Ga-DOTATOC PET and 111In-DTPAOC (Octreoscan) SPECT in patients with neuroendocrine tumours. Eur J Nucl Med Mol Imaging.

[8] Gabriel M, Decristoforo C, Kendler D, Dobrozemsky G, Heute D, Uprimny C (2007). 68Ga-DOTA-Tyr3-octreotide PET in neuroendocrine tumors: comparison with somatostatin receptor scintigraphy and CT. J Nucl Med.

[9] Horton KM, Fishman EK (2003). The current status of multidetector row CT and three-dimensional imaging of the small bowel. Radiol Clin North Am.

[10] Gabriel M, Hausler F, Bale R, Moncayo R, Decristoforo C, Kovacs P (2005). Image fusion analysis of (99m)Tc-HYNIC-Tyr(3)-octreotide SPECT and diagnostic CT using an immobilisation device with external markers in patients with endocrine tumours. Eur J Nucl Med Mol Imaging.

[11] Ruf J, Heuck F, Schiefer J, Denecke T, Elgeti F, Pascher A (2010). Impact of Multiphase 68Ga-DOTATOC-PET/CT on therapy management in patients with neuroendocrine tumors. Neuroendocrinology.

[12] Van Riet J, Rattat D, Verbruggen A, Mortelmans L, Mottaghy FM (2009). Ga-68 DOTATOC PET/CT changed the therapeutic course of a patient with the sudden onset of vision problems. Clin Nucl Med.

[13] Frilling A, Sotiropoulos GC, Radtke A, Malago M, Bockisch A, Kuehl H (2010). The impact of 68Ga-DOTATOC positron emission tomography/computed tomography on the multimodal management of patients with neuroendocrine tumors. Ann Surg.

[14] Van Hoe L, Gryspeerdt S, Marchal G, Baert AL, Mertens L (1995). Helical CT for the preoperative localization of islet cell tumors of the pancreas: value of arterial and parenchymal phase images. Am J Roentgenol.

[15] Stafford-Johnson DB, Francis IR, Eckhauser FE, Knol JA, Chang AE (1998). Dual-phase helical CT of nonfunctioning islet cell tumors. J Comput Assist Tomogr.

[16] Chung MJ, Choi BI, Han JK, Chung JW, Han MC, Bae SH (1997). Functioning islet cell tumor of the pancreas. Localization with dynamic spiral CT. Acta Radiol.

[17] Schreiter NF, Nogami M, Steffen I, Pape UF, Hamm B, Brenner W (2012). Evaluation of the potential of PET-MRI fusion for detection of liver metastases in patients with neuroendocrine tumours. Eur Radiol.

[18] Andersson T, Eriksson B, Hemmingsson A, Lindgren PG, Oberg K (1987). Angiography, computed tomography, magnetic resonance imaging and ultrasonography in detection of liver metastases from endocrine gastrointestinal tumours. Acta Radiol.

[19] Wallace S, Ajani JA, Charnsangavej C, DuBrow R, Yang DJ, Chuang VP (1996). Carcinoid tumors: imaging procedures and interventional radiology. World J Surg.

[20] Ricke J, Hanninen EL, Amthauer H, Lemke A, Felix R (2000). Assessment of the vascularization of neuroendocrine tumors by stimulated acoustic emission of SH U 508A ultrasound contrast agent and color or power Doppler sonography. Invest Radiol.

[21] Zhernosekov KP, Filosofov DV, Baum RP, Aschoff P, Bihl H, Razbash AA (2007). Processing of generator-produced 68Ga for medical application. J Nucl Med.

[22] Prabhakar HB, Sahani DV, Fischman AJ, Mueller PR, Blake MA (2007). Bowel hot spots at PET-CT. Radiographics.

[23] Versari A, Camellini L, Carlinfante G, Frasoldati A, Nicoli F, Grassi E (2010). Ga-68 DOTATOC PET, endoscopic ultrasonography, and multidetector CT in the diagnosis of duodenopancreatic neuroendocrine tumors: a single-centre retrospective study. Clin Nucl Med.

[24] Ruf J, Schiefer J, Furth C, Kosiek O, Kropf S, Heuck F (2011). 68Ga-DOTATOC PET/CT of neuroendocrine tumors: spotlight on the CT phases of a triple-phase protocol. J Nucl Med.

[25] Prasad V, Ambrosini V, Hommann M, Hoersch D, Fanti S, Baum RP (2010). Detection of unknown primary neuroendocrine tumours (CUP-NET) using (68)Ga-DOTA-NOC receptor PET/CT. Eur J Nucl Med Mol Imaging.

